# Atogepant Reduces Psychological Dependence on Acute Treatments Evaluated With the Leeds Dependence Questionnaire: A Prospective Study

**DOI:** 10.1002/brb3.71553

**Published:** 2026-07-23

**Authors:** Luigi Francesco Iannone, Marina Romozzi, Alberto Boccalini, Flavia Lo Castro, Claudia Altamura, Fabrizio Vernieri, Simona Guerzoni

**Affiliations:** ^1^ Department of Biomedical, Metabolic, and Neural Science University of Modena and Reggio Emilia Modena Italy; ^2^ Dipartimento Universitario di Neuroscienze Università Cattolica del Sacro Cuore Roma Italy; ^3^ Divisione di Neurologia, Dipartimento di Neuroscienze, Organi di Senso e Torace Fondazione Policlinico Universitario Agostino Gemelli IRCCS Roma Italy; ^4^ Department of Digital and Predictive Medicine, Pharmacology and Clinical Metabolic Toxicology, Headache Center and Drug Abuse, Laboratory of Clinical Pharmacology and Pharmacogenomics AOU Policlinico di Modena Modena Italy; ^5^ Headache Unit Fondazione Policlinico Universitario Campus Bio‐Medico Roma Italy; ^6^ Department of Medicine and Surgery Università Campus Bio‐Medico Di Roma Roma Italy

**Keywords:** acute treatments, atogepant, dependence‐like, migraine

## Abstract

**Background:**

The Leeds Dependence Questionnaire (LDQ) is a validated tool for assessing psychological dependence across various substances and has been adapted for use in headache patients. No study has yet explored the effects of preventive anti‐CGRP treatments like atogepant on psychological dependence related to acute migraine medications.

**Methods:**

We conducted a prospective, real‐world, single‐center study on patients with migraine who were treated with atogepant 60 mg daily for 12 weeks. LDQ scores were assessed at baseline and after 12 weeks. Monthly headache days (MHDs), acute medication use, and presence of psychiatric comorbidities were collected. A linear mixed‐effects model evaluated the effect of treatment over time, adjusting for medication overuse (MO) status and psychiatric comorbidities.

**Results:**

We included 43 patients (69.8% with chronic migraine, 67.4% with MO). The LDQ total score significantly decreased from 7.95 ± 5.79 to 6.42 ± 5.08 after treatment (mean difference: –1.53, *p* = 0.032). Significant improvements were seen in three specific LDQ items reflecting loss of control, compulsive use, and psychological distress. No significant correlation was found between the reduction in LDQ score and the change in acute medication use. Mixed‐effects modeling confirmed a significant effect of treatment (*p* = 0.022), but independent of MO or MOH status or psychiatric comorbidities.

**Conclusion:**

Preventive treatment with atogepant is associated with a significant reduction in psychological dependence on acute migraine medications, as measured by the LDQ. These findings support the need to assess dependence‐like behavior in clinical migraine care and highlight the potential behavioral benefits of effective preventive treatments.

## 1 Introduction

Migraine is a highly prevalent and disabling neurological condition, affecting approximately one billion people globally (Ashina 2020). It ranks among the leading causes of disability and is often associated with several comorbidities (Burch et al. 2019; Hus⊘y et al. 2025). The misuse and overuse of acute treatments is a frequent and often overlooked issue; it can complicate migraine management and could be related to medication overuse headache (MOH) (Ashina et al. 2023). The misuse of acute treatment has a considerable direct and indirect cost for the healthcare system and a substantial burden, increasing disability and reducing quality of life for patients (Ashina et al. 2023; Diener et al. 2019).

Dependence‐like behaviors, such as the habitual need for acute medications, difficulty in abstaining from use despite adverse effects or poor tolerability, psychological attachment, anticipatory anxiety, or behavioral rituals, have been increasingly reported in migraine patients (Takahashi et al. 2021).

Overall, the Severity of Dependence Scale is the most widely used scale to assess dependence behavior (Gossop et al. 1995; Lau et al. 2020; Takahashi et al. 2021), although other, more detailed, scales are available. The assessment of dependency‐like behaviors via the SDS showed a good correlation and agreement with the LDQ, both in global scores and individual items (Bottiroli et al. 2022).

The Leeds Dependence Questionnaire (LDQ) is a validated tool originally developed to assess psychological dependence in substance use disorders (Raistrick et al. 1994) but has since been adapted to evaluate dependence‐like behavior in patients with headache (Ferrari et al. 2006; Wang et al. 2023).

Prior studies using the LDQ have demonstrated that chronic migraine (CM) patients with medication overuse (MO) could exhibit levels of dependence similar to subjects with substance addiction and that patients with daily headache and MO had more severe dependence behaviors compared with those with episodic migraine (EM), episodic cluster headache, or episodic‐tension type headache (Ferrari et al. 2006). Furthermore, it has been shown that the severity of dependence behaviors was not only correlated with MO in the general population (Grande et al. 2009) but was also predictive of the prognosis of withdrawal (Lundqvist et al. 2012).

Atogepant, a novel oral calcitonin gene‐rerelated peptide (CGRP) receptor antagonist (gepant), has shown efficacy in reducing monthly migraine days (MMDs) and acute medications in both EM and CM patients in randomized controlled trials, regardless of medication overuse (Grande et al. 2009), and in a real‐world setting (Barbanti et al. 2025; Vernieri et al. 2025). The use of anti‐CGRP drugs (gepants and anti‐CGRP monoclonal antibodies [mAbs]) has redefined the treatment of MO/MOH, demonstrating their effectiveness in this population, irrespective of prior withdrawal strategies or other interventions, type of overused analgesics and, although with inconsistent results, other comorbidities (Haghdoost et al. 2023; Pensato et al. 2022; Sánchez‐Rodríguez et al. 2024).

However, no studies to date have assessed whether preventive treatment with atogepant (or any other anti‐CGRP drugs) can reduce the psychological dependence on acute medications as captured by the LDQ in patients with and without MO.

This prospective observational study aimed to evaluate the effect of atogepant 60 mg daily over 12 weeks on psychological dependence on acute medications in migraine patients using the LDQ, and to explore its associations with clinical outcomes such as migraine frequency, acute treatment use, and presence of MO.

## 2 Methods

### 2.1 Study Design

We conducted a real‐world, prospective, single‐center, investigator‐initiated, and independent study involving all consecutive outpatients receiving oral atogepant 60 mg for EM or CM at the Headache Center of Modena General Hospital, Modena, Italy.

All patients who could potentially complete a 12‐week follow‐up were enrolled, regardless of treatment discontinuation, between June and December 2024. Participants who discontinued therapy but completed the LDQ at week 12 were also included. The same cohort was concurrently analyzed as part of the STAR study (Vernieri et al. 2025) and in a parallel project investigating sleep quality and sleep‐related disturbance (Iannone et al. 2025).

The LDQ was administered by a clinician at baseline (T0, prior to starting atogepant) and after 12 weeks (T3), irrespective of treatment continuation. Data were collected using the Research Electronic Data Capture (REDCap) online platform. Atogepant was not reimbursed by the Italian National Health Service during the study period; patients received the medication under an agreement between the Italian Medicines Agency (AIFA), regional healthcare authorities, and the manufacturer, which provided the drug free of charge.

The local Ethics Committee approved the study as part of the Registro Italiano Cefalee (RICe) project (Studio RICe, 14591_oss CEAVC and subsequent amendments). All participants provided written informed consent before treatment initiation.

### 2.2 Patient Features

Patients were enrolled irrespective of the number of preventive treatments previously discontinued due to lack of efficacy or tolerability. Ineffectiveness was defined according to the European Headache Federation (EHF) criteria (Sacco et al. 2022).

***Inclusion criteria*** were: (i) >18 years old; (ii) diagnosis of migraine without aura, with aura, or CM according to ICHD‐3 (Headache Classification Committee of the International Headache Society (IHS) The International Classification of Headache Disorders, Third Edition 2018); (iii) at least 4 monthly migraine days (MMDs) in the 3 months before inclusion in the study; (iv) availability of headache diaries at least one month before inclusion; (v) clinical indication for prescription of atogepant 60 mg; and (vi) stable treatment with other preventive drugs for migraine and sleep.

***Exclusion criteria*** were: (i) patients with any contraindications to atogepant according to the summary of product characteristics; (ii) pregnancy and breastfeeding; (iii) cognitive decline; (iv) intellectual disability; and (v) use of recreational drugs.

### 2.3 Collected Variables

The following demographic and clinical characteristics were collected: age, sex, concomitant and previous preventive treatments, monthly headache days (MHDs), number of monthly acute medications (AMNs), days with at least one use of acute medications (AMDs) before atogepant first intake (i.e., baseline). We further calculated the ratio between AMNs and MHDs (i.e., AMNs/MHDs) at baseline and 12 weeks to evaluate further the impact of the absolute number of analgesics on this coefficient. Medication‐overuse headache (MOH) was defined according to ICHD‐3 criteria. We defined patients with MO (and not MOH) as those with overuse of acute or symptomatic headache medication (on 10 or more days/month for triptans and combination analgesics, or 15 or more days/month for non‐opioid analgesics), regardless of diagnosis, to better underscore the role of overuse. Furthermore, we included patients with and without MO to assess the behavioral dependence in the overall population, reflecting the clinical practice, and to assess the impact of MO status at baseline.

To evaluate the overall burden of migraine, we defined MHDs as headache days—that is, any day on which a patient recorded a headache. Clinical variables not related to migraine, including comorbidities, were reported according to patients’ charts and outpatient interviews during clinical practice.

Finally, as part of the STAR study and relevant to this analysis, a panel of questionnaires for migraine disability was administered at baseline and after 12 weeks of treatment (T3): the Headache Impact Test (HIT‐6) (Martin et al. 2004), the Migraine Disability Assessment (MIDAS) questionnaire (D'Amico et al. 2001), the 12‐item Allodynia Symptom Checklist (ASC‐12) (Lipton et al. 2008), and the Migraine Interictal Burden Scale (MIBS‐4) (Buse et al. 2007). The Patient's Global Impression of Change (PGIC) was administered at T3 (Hurst and Bolton 2004). Any AEs were collected during the study.

### 2.4 Leeds Dependence Questionnaire (LDQ)

We administered the validated LDQ questionnaire, modified for headache by Ferrari et al. (2006) to assess dependence on acute migraine treatment at baseline (T0) and after 12 weeks of treatment (T3). The questionnaire questions are reported (along with results) in Table 1.

**TABLE 1:** Change in single subitems of the LDQ total score after 12 weeks of treatment with atogepant.

LDQ items		(Mean [95% CI])		*Z*	*p*‐value
		Baseline	12 weeks		
1	Do you often think about when you will be able to take an analgesic?	1.09 (0.7–1.1)	0.81 (0.5–1.0)	−2.276	**0.023**
2	Is taking analgesics more important than anything else you could do during the day?	0.63 (0.3–0.9)	0.47 (0.2–0.6)	−1.355	0.176
3	Do you feel your need to take analgesics is too difficult to control?	0.58 (0.3–0.8)	0.35 (0.1–0.5)	−2.500	**0.012**
4	Do you organize your days around when you will take analgesics?	0.35 (0.1–0.5)	0.26 (0.1–0.4)	−1.100	0.271
5	Do you take analgesics in a specific way to increase their effect?	0.49 (0.2–0.7)	0.44 (0.2–0.6)	−0.577	0.564
6	Do you take analgesics in the morning, afternoon, and evening?	0.81 (0.5–1.1)	0.6 (0.3–0.8)	−1.897	0.058
7	Do you think you will have to continue taking analgesics once you have started?	0.77 (0.4–1.0)	0.65 (0.4–0.8)	−1.073	0.283
8	Is the effect more important than the type of analgesic used?	1.0 (0.7–1.3)	1.07 (0.7–1.3)	−0.319	0.750
9	Do you want to take more analgesics when the effect starts to wear off?	0.72 (0.5–0.9)	0.58 (0.3–0.8)	−1.126	0.260
10	Do you find it difficult to cope with life without analgesics?	1.51 (1.2–1.8)	1.19 (0.9–1.4)	−2.645	**0.008**

*Note*: Values in bold are statistically significant; *p* values calculated with Wilcoxon test.

The LDQ is a self‐administered, 10‐item survey that uses a 4‐point scale (0 = never, 1 = sometimes, 2 = often, 3 = nearly always) to assess the frequency of dependence‐related behaviors (Raistrick et al. 1994). It was developed to evaluate the severity of psychological dependence across a wide range of substances, regardless of type or amount, and is designed to be sensitive to changes over time. The LDQ has been previously validated in populations with alcohol and opioid use, particularly within addiction and psychiatric settings (Ferrari et al. 2006). Notably, there is no established cut‐off score to define dependence, but a recent study defined a ≥7 cut‐off to detect patients with MOH (Wang et al. 2023). For the present study, the original wording was adapted to refer specifically to “analgesic/s” instead of alcohol or drugs in order to ensure relevance for headache patients (Corbelli et al. 2012; Ferrari et al. 2006), and we did not use cut‐offs.

### 2.5 Outcomes and Analysis

The primary outcome was the change in the LDQ total score from before treatment (T0) to 12 weeks after treatment (T3) with atogepant.

**Secondary outcomes included from baseline to T3**:

- Changes in the LDQ ten subitems.
- Correlation of the LDQ questionnaire score with change in acute medication use (both AMNs and AMDs).
- Correlation of the LDQ questionnaire score with change in MHDs.

### 2.6 Statistical Analysis

No formal sample size calculation was performed due to the exploratory nature of the study and lack of prior real‐world data on gepants and dependence. The Shapiro–Wilk test indicated non‐normal distribution for several variables; thus, non‐parametric tests were applied. Continuous variables were expressed as mean (95% CI, IQR, or SD as appropriate), and categorical variables as number (percentage). Missing data was not imputed and are reported in the text and tables.

Pre–post comparisons for continuous variables were analyzed using the Wilcoxon signed‐rank test; the exact McNemar test was used for paired proportions. Between‐group comparisons of continuous variables were conducted using the Mann–Whitney U test. Correlations between LDQ score changes and migraine‐related variables were evaluated using Spearman's rank correlation coefficient (rho).

To evaluate the potential influence of two co‐variables (medication overuse and psychiatric comorbidities) that are demonstrated to strongly influence LDQ score (and overall dependence‐like behavior) in migraine patients, we performed a linear mixed‐effects model (LMM) in the first model using MO status and in the second model using MOH diagnosis. Medication overuse/MOH diagnosis and psychiatric comorbidities were selected a priori because previous literature consistently identified them as major contributors to dependence‐like behaviors in migraine patients. Additional predictors were intentionally not included to reduce overfitting risk and unstable estimates given the limited sample size.

LMMs were performed as exploratory sensitivity analyses to evaluate whether longitudinal changes in LDQ total score were influenced by clinically relevant covariates while accounting for repeated within‐subject observations. Separate models were conducted including medication overuse status or MOH diagnosis, together with psychiatric comorbidity.

The dataset was structured with each participant contributing two observations (baseline and 12‐week follow‐up). Time was modeled as a within‐subject factor, while MO/MOH status and psychiatric comorbidity were treated as between‐subject categorical fixed effects. Interactions between time and the between‐subject variables were included to explore potential differential changes over time.

To account for inter‐individual variability, a random intercept for subject (ID) was included in the model. The repeated‐measures structure was modeled using a compound symmetry (CS) covariance matrix, which is appropriate for two equally spaced time points. Models were estimated using restricted maximum likelihood (REML), and model fit was evaluated using the restricted log‐likelihood. Residual distributions and model assumptions were visually inspected and did not reveal major violations preventing model estimation.

Given the relatively small sample size and the non‐normal distribution of some variables, findings from the LMM analyses should be interpreted cautiously.

A two‐tailed *p*‐value <0.05 was considered significant for all variables, with a Bonferroni's correction where appropriate. All data were analyzed using SPSS software version 29.0 (IBM Corp. SPSS Statistics, Armonk, NY, USA) and graphs designed using GraphPad Prism version 10.00 (La Jolla, USA).

## 3 Results

The final study population included 43 participants (93.0% females, age of 51.6 [IQr 48.4–54.8] years, age at disease onset of 18.9 [16.0–21.7] years). Thirty (69.8%) participants had CM, 29 (67.4%) had MO, and 23 (53.5%) had a diagnosis of MOH. Figure S1 reports the flow chart of the study.

At baseline, participants presented a mean of 20.5 (8.2) MHDs, a mean of 18.3 (7.9) AMDs, and 24.0 (15.9) AMNs. Clinical and demographic features are reported in Table 2. Migraine‐related variables are fully detailed in Table S1. The average number of previously failed preventive classes was 2.7 [2.3–3.2]. Among them, 27 patients (62.8%) had previously received treatment with anti‐CGRP monoclonal antibodies (mAbs). Previous preventive treatments are detailed in Table S1.

**TABLE 2:** Clinical and demographic features of the overall population.

	Cohort (*n* = 43)
Age years; mean (SD)	51.7 (10.3)
Female sex, % (*n*)	93.0 (40)
Onset age years; mean (SD)	18.9 (8.8)
CM, *n* (%)	30 (69.8)
MO, *n* (%)	29 (67.4)
Age of chronicization; mean (SD)^a^	35.3 (9.2)
BMI kg/m^2^; mean (SD)	23.2 (4.2)
Weight kg; mean (SD)	62.1 (12.8)
Pain intensity (NRS); mean (SD)	7.7 (0.9)
Clinically relevant comorbidities, % (*n*)	53.5 (23)

^a^ Calculated in patients with CM. Percentages are expressed on column total.

CM, chronic migraine; MO, medication overuse; BMI, body mass index; SD, standard deviation.

Twenty‐three participants (53.5%) had at least one comorbidity (Table S1), and among them, 9 (20.9%) had psychiatric comorbidity (anxiety or depression). Only two subjects (4.7%) dropped out of the treatment with atogepant due to adverse events (i.e., nausea) and poor tolerability. The overall effectiveness and tolerability of atogepant is reported in Supplementary data.

### 3.1 LDQ Questionnaire Total Score

**FIGURE 1:**
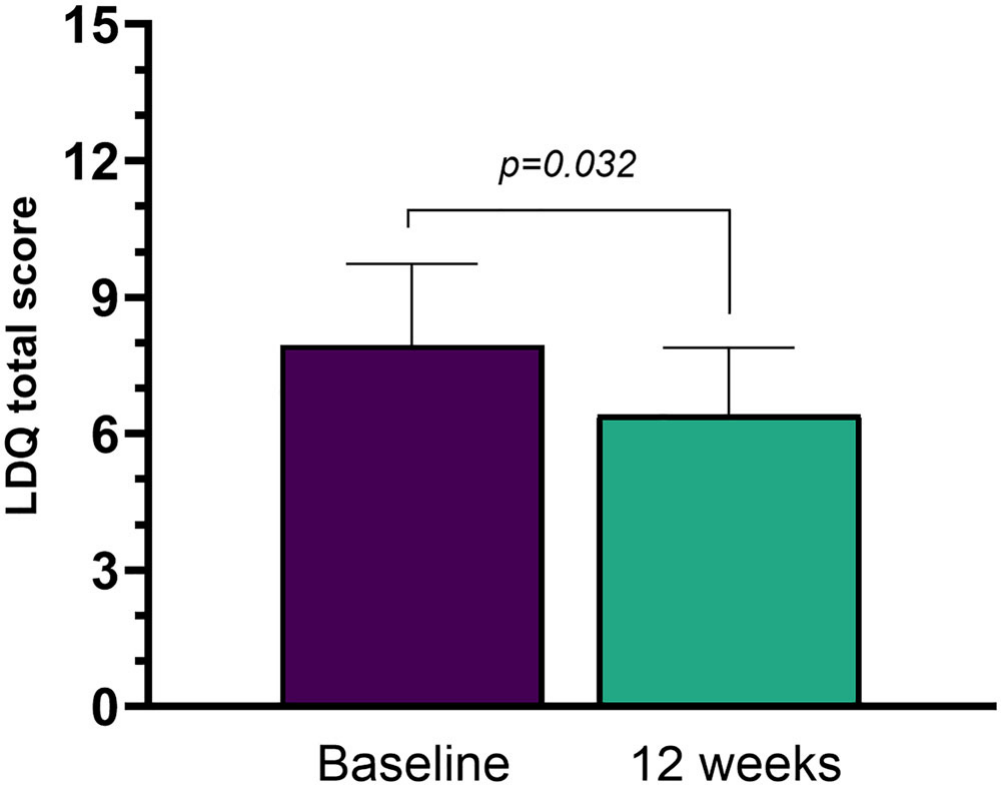
LDQ total score, pre‐ and post‐treatment (12 weeks) with atogepant. Statistical comparisons performed using Wilcoxon signed‐rank test. LDQ, Leeds Dependence Questionnaire.

At baseline, the mean LDQ total score was 7.95 ± 5.79 (95% CI: 6.17–9.74), with a median of 7.0, indicating moderate psychological dependence on acute migraine medications. After 12 weeks of preventive therapy, the mean LDQ score decreased to 6.42 ± 5.08 (95% CI: 4.85–7.98), with a median of 6.0 (Figure 1 and Table 2).

The mean change in LDQ total score was –1.53 ± 4.10, with a 95% CI from –2.80 to –0.27. Despite the moderate absolute reduction, the Wilcoxon signed‐rank test showed a statistically significant improvement in dependence scores (*Z* = –2.13, *p* = 0.032), with a moderate effect size (*r* = 0.32) (Table 3). Notably, 20 patients (46.5%) showed a decrease in LDQ score, 11 (25.6%) showed an increase, while 12 (27.9%) had unchanged scores.

**TABLE 3:** Change in LDQ total score after 12 weeks of treatment with atogepant.

	Mean (SD)	95% CI	Median	*p* value
**Baseline**	7.95 (5.79)	6.17 to 9.74	7.00	**0.032**
**12 weeks**	6.42 (5.08)	4.85 to 7.98	6.00	
**Mean change**	−1.53 (4.10)	−2.80 to −0.27	—	—

*Note*: Values in bold are statistically significant; *p* values calculated with Wilcoxon test.

### 3.2 LDQ Questionnaire Item‐Level Scores

**FIGURE 2:**
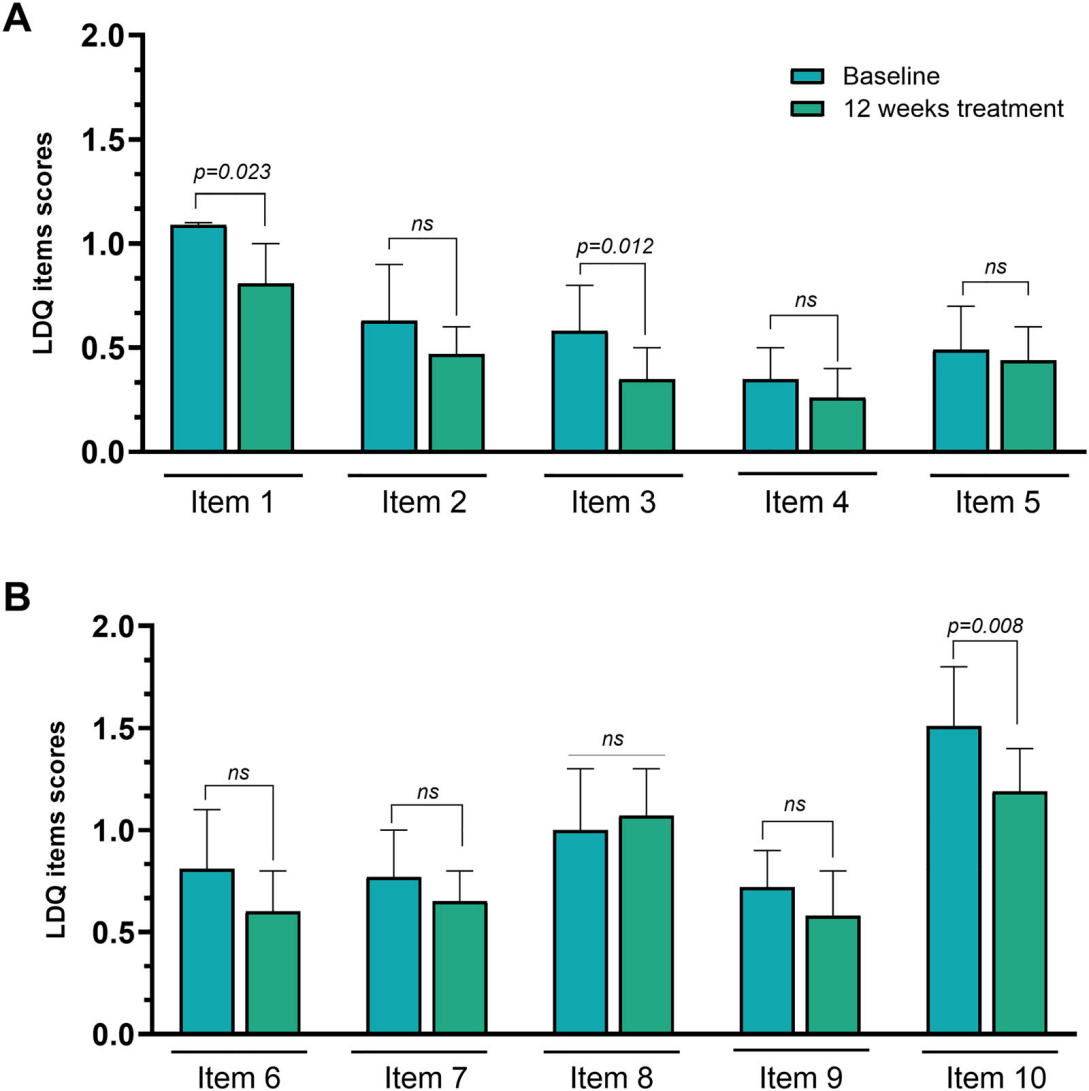
LDQ 10 individual items (panels A [first five items] and B [last five items]) at baseline and after 12 weeks with atogepant. Statistical comparisons performed using Wilcoxon signed‐rank test (uncorrected). *Ns, not significant. LDQ; Leeds Dependence Questionnaire*.

At the item level, patients reported lower scores across nearly all domains of the LDQ following 12‐week treatment with atogepant than at baseline. Statistically significant improvements were observed in several key items (Table 1 and Figure 2).

Specifically, the item “*Do you often find yourself thinking about when you will next be able to take an analgesic?*” showed a significant decrease (*Z* = –2.27, *r* = 0.34, *p* = 0.023), with the mean dropping from 1.09 to 0.81. Similarly, dependence‐related loss of control, as assessed by “*Do you feel your need for analgesics is too difficult to control?*” significantly improved (*Z* = –2.50, *r* = 0.38, *p* = 0.012). Finally, patients were significantly less likely to endorse the item “*Do you find it difficult to cope with life without analgesics?*” after treatment (*Z* = –2.64, *r* = 0.40, *p* = 0.008).

Although other items did not reach statistical significance, they showed trends toward reduction. These included beliefs regarding the centrality of analgesics to daily life, behavioral organization around intake, and compensatory strategies to enhance analgesic effects. All items are reported in Table 3.

### 3.3 Psychiatric Comorbidities and Medication Overuse Influence

In the LMM, there was a significant main effect of time on LDQ total scores (*F*(1, 40) = 5.66, *p* = 0.022), confirming a statistically significant reduction in dependence‐like behaviors after 12 weeks of treatment with atogepant.

There was a trend‐level effect for MO status (*F*(1, 40) = 3.84, *p* = 0.06) and MOH diagnosis ((*F*(1, 40) = 2.50, *p* = 0.122), suggesting higher LDQ scores in patients with MO and MOH, though this did not reach statistical significance. The interaction between time and MO status/MOH diagnosis was also not significant (*F*(1, 40) = 0.08, *p* = 0.779) and (*F*(1, 40) = 0.52, *p* = 0.474), respectively, indicating that the reduction in LDQ scores over time was similar in both patients with and without MO/MOH.

No significant effects were observed for psychiatric comorbidity (*F*(1, 40) = 2.98, *p* = 0.09) or its interaction with time (*F*(1, 40) = 1.03, *p* = 0.31).

### 3.4 Migraine‐Related Variables Correlation With LDQ Total Score

The analysis did not demonstrate a significant correlation between the reduction in LDQ total score and the reduction in the use of acute migraine medications, either in terms of AMNs or AMDs. Specifically, LDQ score change was not significantly correlated with AMNs reduction (rho = –0.211, *p* = 0.17) or AMDs reduction (rho = 0.017, *p* = 0.91), nor with reductions in MHDs (rho = –0.014, *p* = 0.92). Furthermore, we did not find any correlation between the ratio AMNs/MHDs and LDQ score chance (rho = –0.163, *p* = 0.40).

As expected, the strong intercorrelations observed between migraine clinical variables, especially between AMDs and both MHDs (rho = 0.423, *p* < 0.005) and AMNs (rho = 0.796, *p* < 0.001), support the internal consistency of clinical improvement. All variables pre–post treatment are reported in Table S2.

## 4 Discussion

In this prospective real‐world study, 12‐week treatment with atogepant 60 mg daily significantly reduced psychological dependence on acute migraine treatments, as measured by the LDQ. This is the first evidence, to our knowledge, suggesting that preventive therapy with an anti‐CGRP drug may not only reduce acute medication intake but also mitigate the underlying behavioral dependence associated with acute treatment use. Notably, atogepant treatment led to significant decreases in key LDQ subitems reflecting psychological distress, compulsive use, and functional impairment associated with medication intake.

Furthermore, we did not find any statistically significant effect of MO status and MOH diagnosis, although a trend was observed, suggesting higher LDQ scores in patients with MO and MOH diagnosis, as expected. The interaction between time and MO status/MOH diagnosis was also not significant, indicating that the reduction in LDQ scores over time was similar in these two groups of patients. No significant effects were observed also for psychiatric comorbidities.

Interestingly, no statistically significant direct correlations were found between the reduction in LDQ score and the reduction in acute medication use (AMNs and AMDs) and MHDs, although both LDQ score and acute medications significantly decreased post‐treatment, clearly indicating an improvement during atogepant therapy. The relationships among AMNs, AMDs, and MHDs reflect the expected cascade whereby fewer headache days lead to fewer occasions requiring acute medication, and consequently, fewer days of actual medication intake.

However, behavioral and psychological dependence may respond to treatment independently of medication frequency, as suggested by the lack of a direct correlation between LDQ scores, medication intake, and MO/MOH status.

Interestingly, the baseline mean LDQ score of our cohort (7.9 ± 5.7) was close to the cutoff value of ≥7 proposed by Wang et al. (Ferrari et al. 2006; Wang et al. 2023) for identifying MOH‐related dependence behaviors, further supporting the clinical relevance of dependence‐like symptoms in our population. However, despite the significant reduction in both LDQ scores and migraine‐related variables, these changes were not significantly correlated. This finding may suggest that behavioral dependence‐like features and symptomatic improvement are not fully synchronous processes. Dependence‐related behaviors may partially persist despite clinical improvement or, conversely, may improve through enhanced perceived control, reduced anticipatory anxiety, or adaptive coping strategies even before substantial reductions in acute medication intake occur.

Thus, patients may have experienced changes in illness perception, coping behaviors, or perceived control over their condition. Accordingly, the observed improvement during atogepant treatment may reflect a shift in dependence‐like patterns not directly associated with the magnitude of reduction in acute medication use or headache frequency. Longer treatment durations may further influence these relationships and potentially reveal stronger longitudinal associations, which should be assessed in future studies.

Overall, medication dependence in migraine may not be solely a function of quantity or frequency, but also of psychological attachment, anticipatory anxiety, or behavioral ritual, all factors potentially improved through enhanced disease control and/or improved quality of life (Biagianti et al. 2012; Corbelli et al. 2012; Lau et al. 2020; Takahashi et al. 2021).

Previous studies have established that chronic migraine patients with high acute medication use/overuse (with or without MO/MOH) often demonstrate dependency patterns similar to those observed in substance use disorders (Ferrari et al. 2006; Wang et al. 2023). In a study on 445 patients with MOH, 313 (69%) presented substance dependence according to the DSM‐IV criteria (Bottiroli et al. 2022).

However, the distinction between medication use/overuse in migraine and substance addiction is critical when interpreting dependence‐like behaviors in migraine patients. While the high use of acute treatments (with or without MO) and addiction may share certain behavioral patterns, such as frequent medication intake, psychological preoccupation, and difficulty in discontinuation, the underlying mechanisms and clinical contexts differ substantially. A recent review outlined all similarities and differences (Takahashi et al. 2021). Addiction is typically characterized by compulsive drug‐seeking behavior driven by reward, craving, and loss of control, often emerging in recreational or hedonic settings (Everitt and Robbins 2005; Leyton 2007). Conversely, medication use/overuse in migraine is a maladaptive coping strategy in response to chronic or frequent pain, where the repeated use of analgesics or acute treatments is primarily aimed at symptom relief rather than euphoria or escape (Ashina et al. 2023). Furthermore, patients with MOH tend to have peculiar profile, including higher levels of alexithymia, suggesting that they tend have high levels of negative emotions and maladaptive coping strategies that may lead to overuse (Migliore et al. 2020; Romozzi et al. 2022).

Importantly, migraine patients often lack the core features of substance use disorder, such as intoxication, craving, or drug‐seeking per se. Many patients are distressed by their medication use and show improvement after withdrawal and/or appropriate preventive therapy (Diener et al. 2019; Diener et al. 2022). These substantial differences may justify the reason why some items in our study are significantly different. However, neurobiological overlaps have been described. For instance, both conditions involve the dopaminergic reward system (Akerman and Goadsby 2007; Everitt and Robbins 2005; Leyton 2007), and psychiatric comorbidities such as anxiety and depression are frequent in both populations, possibly facilitating the reinforcement of maladaptive behaviors (Minen et al. 2016; Shantna et al. 2009). Interestingly, we did not find a correlation between LDQ score improvement and psychiatric comorbidities. Finally, some patients with migraine may exhibit more pronounced dependence‐like features, such as loss of control and continued use despite awareness of harm, blurring the boundaries with addiction (Calabresi and Cupini 2005).

Thus, medication use/overuse in migraine and addiction should not be mixed, an understanding of their differences and overlaps is necessary. Recognizing that some migraine patients may lie along a continuum of behavioral vulnerability allows for more individualized approaches to treatment and supports the utility of tools like the LDQ to explore these aspects.

This is the first study to report the impact of atogepant, and anti‐CGRP drugs, on psychological dependence to acute treatments. The strengths of our study include its prospective design, which reflects common clinical practice in tertiary headache centers, and the inclusion of a migraine patient population often excluded from RCTs. Another strength is the use of a validated and reproducible questionnaire. Furthermore, we adjusted our analysis with a mixed effect model for psychiatric comorbidities and MO, two main variables involved in acute medication use, and correlated LDQ score with migraine‐related variables.

However, our study has several limitations, including the lack of a control group, the small sample size, and reliance on self‐report measures, which may limit generalizability. Furthermore, supporting questionnaires such as the SDS could be useful for internal validation of LDQ and, unfortunately, we did not collect socioeconomic status, a variable that can affect acute medication use and misuse. Additionally, the study duration was limited to 12 weeks, and longer follow‐ups are needed to assess the sustained impact on psychological behavior improvement. Despite these limitations, the findings provide evidence supporting the role of CGRP‐targeted therapies in reducing not only migraine burden but also behavioral complications such as medication dependence.

## 5 Conclusions

Preventive treatment with atogepant in a real‐world setting is associated with reduced psychological dependence on acute medications. These findings suggest a potential role for gepants in the treatment of patients with MO/MOH and support a potential broader use of the LDQ in routine headache care.

## Author Contributions

**Claudia Altamura**: supervision, visualization, validation. **Alberto Boccalini**: data curation, validation, methodology, visualization. **Fabrizio Vernieri**: supervision, visualization, validation. **Marina Romozzi**: software, formal analysis, writing – review and editing, visualization. **Flavia Lo Castro**: methodology, validation, visualization, data curation. **Luigi Francesco Iannone**: conceptualization, investigation, writing – original draft, methodology, validation, visualization, writing – review and editing, project administration, software, formal analysis, data curation, supervision, resources. **Simona Guerzoni**: visualization, validation, resources.

## Funding

The authors have nothing to report.

## Ethics Statements

The local Ethics committee approved the study as part of the Registro Italiano Cefalee (RICe) study (Studio RICe, 14591_oss CEAVC Studio RICe, 14591_oss and subsequent amendments).

## Conflicts of Interest

LFI received financial support, honoraria for scientific lectures and presentations, consulting fees for the participation in advisory boards and support for attending meetings from: Teva, Eli Lilly, Lundbeck, Pfizer, Organon and AbbVie; he is Associate Editor for Frontiers in Neurology and junior Editor for Cephalalgia and Cephalalgia reports. FV has received financial support from Allergan‐AbbVie, Angelini, and Lundbeck for investigator‐initiated trials; consulting fees for the participation in advisory boards from AbbVie, Angelini, Eli Lilly, Lundbeck, Organon, Novartis, Pfizer, and Teva; honoraria from AbbVie, Eli Lilly, Lundbeck, Novartis, Organon, Pfizer, and Teva; support for attending meetings from Abbvie, Amgen, Eli Lilly, Lundbeck, Pfizer, and Teva; he has been Principal Investigator in clinical trials sponsored by AbbVie, Eli Lilly, Lundbeck, Pfizer, and Teva; he is Co‐Specialty Editor for Frontiers in Neurology Headache and Neurogenic Pain section. SG has received fees and honoraria for advisory boards, speaker panels, or clinical investigation studies from Novartis, Teva, Eli Lilly, Pfizer, Lundbeck, Organon, Orion, Angelini, and AbbVie. CA is Associate Editor for Frontiers of Human Neuroscience and Frontiers in Neurology Headache and Neurogenic Pain section; she received travel grants and/or personal fees for advisory boards and speaker panels, from Novartis, Eli‐Lilly, Lundbeck, Teva, Lusofarmaco, Laborest, Abbvie/Allergan, Almirall, and Pfizer. Other authors have no relevant financial or non‐financial interests to disclose.

## Supporting information




**Supplementary Materials**: brb371553‐sup‐0001‐SuppMat.docx

## Data Availability

Data supporting the findings in the present study are reported in the article and in the supplementary materials. The data collected and analyzed for the current study are available from the corresponding author on reasonable request.
