# Supplementary material for: Atogepant Reduces Psychological Dependence on Acute Treatments Evaluated With the Leeds Dependence Questionnaire: A Prospective Study
Source: Brain Behav. 2026 Jul 23;16(7):e71553. doi: 10.1002/brb3.71553 (PMC13396879; doi:10.1002/brb3.71553)
Supplement: Supplementary file 1 — Supplementary Materials: brb371553‐sup‐0001‐SuppMat.docx [file BRB3-16-e71553-s001.docx]

| **Table S1.** Comorbidities and concomitant and prior preventive treatments. | |
| --- | --- |
|  | **Cohort (n=43)** |
| **Clinically relevant comorbidities,** % (n) | 53.5 (23) |
| *Vascular* | 25,6 (11) |
| *Psychiatric* | 20.9 (9) |
| *Gastroenterological* | 14.0 (6) |
| *Neurological* | 4.7 (2) |
| *Immunological* | 14.0 (6) |
| *Endocrinological* | 20.9 (9) |
|  | |
| **Patients with concomitant preventive treatment**, n (%) | 19 (44.2) |
| **Previous ineffective treatments**, median (IQr) [min-max] | 5.05 (3) [4.4 – 5.7] |
| **Drug Classes**, n (%) |  |
| *Beta-blockers* | 31 (72.1) |
| *Tricyclic antidepressant* | 40 (93.0) |
| *Calcium channel blockers* | 23 (53.5) |
| *Antiseizure medications* | 30 (69.8) |
| *SSRIs/SNRIs* | 17 (39.5)/22 (51.2) |
| *Angiotensin receptor antagonists* | 2 (4.7) |
| *OnabotulinumtoxinA* | 23 (53.5) |
| *Anti-CGRP/R mAbs* | 27 (66.8) |
| *CGRP, calcitonin gene related peptide; IQr, interquartile range; SD, standard deviation; SSRI, selective serotonin reuptake inhibitors; SNRI, serotonin-norepinephrine reuptake inhibitor. Percentages are expressed on column total.* | |

**Atogepant reduces psychological dependence on acute treatments evaluated with the Leeds dependence questionnaire (LDQ): a prospective observational study**

*Supplementary data*

| **Table S2** Migraine-related variables and patient reported outcomes and at baseline and after 12-week therapy with atogepant. | | | | |
| --- | --- | --- | --- | --- |
| Mean (SD) | **T0** | **T3** | **Change** (mean, 95%CI) | **p** |
| **MHDs** | 20.5 (8.2) | 10.1 (9.3) | -10.3 (-12-8, -7.8) | **<0.001** |
| **AMNs** | 24.0 (15.9) | 12.2 (15.7) | -11.8 (-16.0, -7.6) | **<0.001** |
| **AMDs** | 18.3 (7.9) | 10.1 (9.6) | -8.3 (-10.5, -5.9) | **<0.001** |
| **MIDAS** | 69.1 (51.8) | 32.7 (39.0) | -44.9 (-63.7, -26.1) | **<0.001** |
| **HIT-6** (n=41) | 64.8 (8.3) | 51.9 (11.7) | -15.3 (-19.0, -11.6) | **<0.001** |
| **ASC-12** (n=42) | 6.2 (4.9) | 2.1 (2.7) | -3.2 (-4.6, -1.7) | **<0.001** |
| *Percentages are expressed on column total.; MHDs, monthly headache days; AMNs, number analgesics per month; AMD; days with at least one analgesics use per month; HIT6, headache impact test; MIDAS, Migraine Disability Assessment questionnaire; SD, Standard deviation.* | | | | |

| **Table S3.** Treatment emergent adverse events, intensity and drop-out rates after 12 weeks of treatment with atogepant. | |
| --- | --- |
|  | **Total Cohort (n=43)** |
| **Patients with at least one treatment-emergent adverse events**, n (%) | 22 (51.2) |
| **Number of adverse events** (n=22), n (%) |  |
| *Constipation* | 16 (37.2) |
| *Lack of appetite* | 8 (18.6) |
| *Nausea* | 4 (9.3) |
| *Fatigue* | 2 (4.7) |
| **AEs intensity**, n (%)^a^ |  |
| *Mild* | 18/20 (90.0) |
| *Intermediate* | 1/20 (5.0) |
| *Severe* | 1/20 (5.0) |
| **Dropout rate**, n (%) | 2 (4.6) |
| *^a^Calculated on 20 adverse events. Percentages are expressed on column total if not otherwise specified.* | |

**Migraine related variables**

A statistically significant improvement was observed across all migraine-related metrics. Monthly headache days (MHDs) decreased by a mean of 10.3 days (95% CI: -12.8 to -7.8; *p* < 0.001) after 12 weeks of treatment. A ≥50% reduction in MHDs (RR50%) was achieved by 67.4% (29/43) of participants, while 39.6% (17/43) experienced a ≥75% reduction. Notably, 4.7% of patients (2/43) achieved complete migraine remission at week 12.

Analgesic use also declined significantly: acute medication numbers (AMNs) were reduced by 11.8 (95% CI: -16.0 to -7.6; *p* < 0.001), and acute medication days (AMDs) by 8.3 (95% CI: -10.5 to -5.9; *p* < 0.001). The proportion of patients meeting criteria for medication overuse headache (MOH) dropped significantly from 53.5% (23/43) at baseline to 39.5% (17/43) at 12 weeks (*p* < 0.001).

Disability and headache-related burden also improved significantly. The MIDAS score decreased by a mean of 44.9 points (95% CI: -63.7 to -26.1; *p* < 0.001), while HIT-6 scores were reduced by 15.3 points (95% CI: -19.0 to -11.6; *p* < 0.001). Patient-reported allodynia, assessed using the ASC-12 scale, showed a mean reduction of 3.2 points (95% CI: -4.6 to -1.7; *p* < 0.001). The Patient Global Impression of Change (PGIC) score at 12 weeks averaged 5.42 (SD = 1.72).

**Overall tolerability and adverse events analysis**

Adverse events (AEs) were reported by 51.2% (22/43) of participants. Most AEs were mild and self-limiting, with only five individuals reporting more than one event. Approximately 90% of all AEs were classified as mild. Two AEs (both nausea) were of moderate to severe intensity and led to treatment discontinuation.

The most frequently reported AEs included constipation (37.2%, 16/43), reduced appetite (18.6%, 8/43), and nausea (9.3%, 4/43).


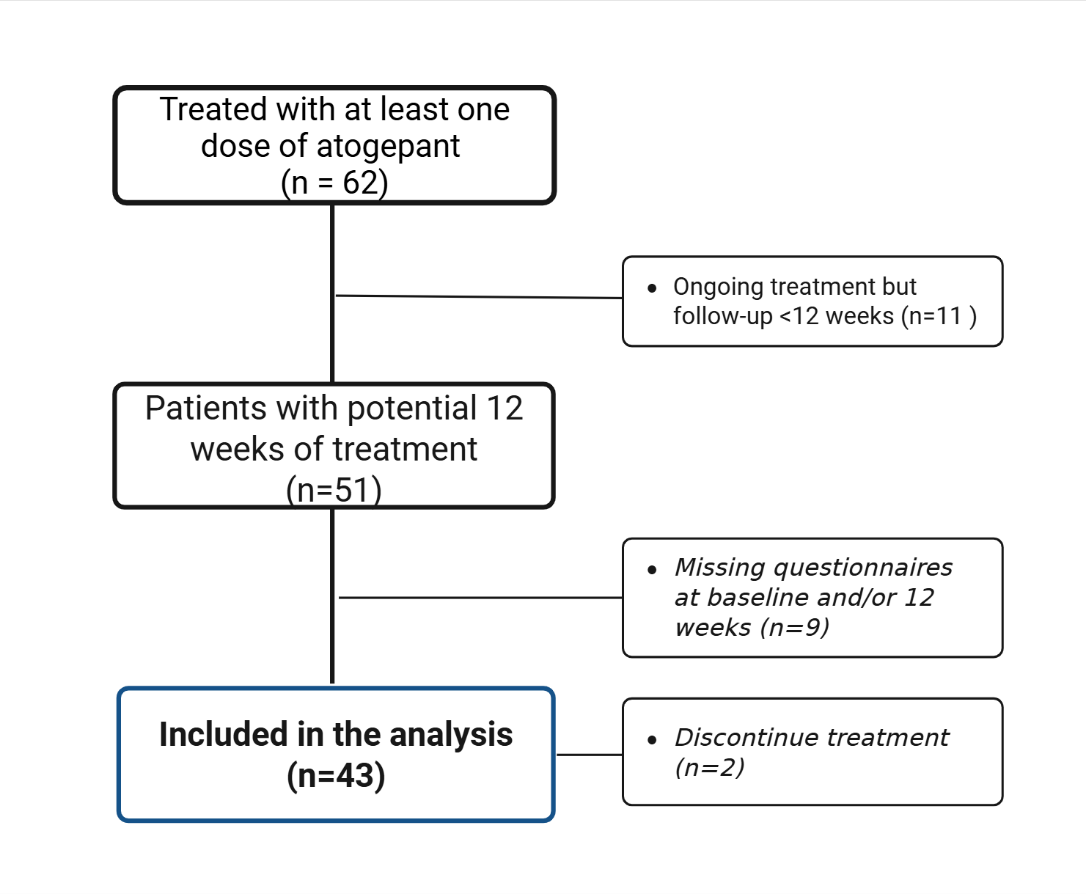


**Figure S1.** Flowchart of patients.
